# Safety Pharmacological Evaluation of the Coffee Component, Caffeoylquinic Acid, and Its Metabolites, Using Ex Vivo and In Vitro Profiling Assays

**DOI:** 10.3390/ph12030110

**Published:** 2019-07-17

**Authors:** Yuto Amano, Hiroshi Honda, Yuko Nukada, Naohiro Ikeda, Masayuki Yamane, Koji Nakano, Akiyo Kameyama, Osamu Morita

**Affiliations:** 1 R&D Safety Science Research, Kao Corporation, 2606 Akabane, Ichikai–Machi, Haga–Gun, Tochigi 321–3497, Japan; 2 Drug Safety Testing Center Co., Ltd., 88-75 Shingou, Higashimatsuyama-shi, Saitama 355-0071, Japan

**Keywords:** safety pharmacology, chlorogenic acid, caffeoylquinic acid, coffee, adverse effects, bioactive compounds, cardiovascular system, central nervous system, gastrointestinal system

## Abstract

Although coffee components have gained interest for use as pharmaceuticals, little is known about their safety pharmacological effects. Hence, we aimed to evaluate the safety pharmacological effects of a chlorogenic acid (CGA)-related compound contained in coffee, 5-*O*-caffeoylquinic acid (5-CQA), and its metabolites, 5-*O*-feruloylquinic acid (5-FQA), caffeic acid (CA), and ferulic acid (FA). Langendorff perfused heart assay, electrophysiological assay of acute rat hippocampal slices, and in vitro Magnus assay of gastrointestinal tracts were conducted at 1–100 µM. Moreover, in vitro profiling assays against 38 major targets were conducted. In the Langendorff assay, no significant adverse effects were observed. In the electrophysiological assay, although epileptiform discharge rates were increased at 10 µM CA with 4-aminopyridine, and area under the curve (AUC) and number of population spike were increased at 10 µM FA with bicuculline, dose dependency was not confirmed, and no significant changes were observed at 1 µM and by CGAs alone. In the Magnus assay, a slight increase in contraction activity was observed at >1 µM FA in the stomach fundi and 100 µM 5-CQA in the ileum, suggesting enterokinesis promotion. No significant interactions were observed in the in vitro profiling assays. Therefore, CGAs could have a fundamental function as safe pharmaceuticals.

## 1. Introduction

Recently, the focus on extending life expectancy has been increasing. Hence, more health foods and supplements containing bioactive ingredients extracted from various herbs and foods are being consumed. Such consumption can expose individuals to active ingredients beyond the daily uptake experience, which might lead to adverse effects. In fact, some clinical toxic effects caused by supplements have been reported recently [1]. For example, synephrine, which is synthesized from *Citrus aurantium*, has been consumed as a supplement for weight-loss; however, it could lead to cardiovascular side effects by binding to the adrenergic receptor. In addition, caffeine was reported to cause arrhythmia when consumed in excess [2]. Therefore, even if such bioactive ingredients are consumed as foods for a long time, an unexpected pharmacological effect could occur by increasing their internal/external exposure or varying their disposition.

Traditional safety assessments of food ingredients including systemic toxicity tests and genotoxicity tests are conducted if there is insufficient food intake experiments and toxicological information [3]. In the development of pharmaceuticals, safety pharmacological evaluation is also necessary to determine the pharmacodynamic effects on cardiovascular function and neuronal activity [ICH-7]. Therefore, safety pharmacological evaluation for bioactive ingredients in health foods is needed to prevent adverse events when developing pharmaceuticals.

Coffee and its components can be considered as examples that should be evaluated for safety pharmacology as coffee has been widely used as beverages and some of its components have pharmacological effects. Some cohort studies reported that habitual drinkers of coffee have a low risk of high blood pressure [4], cardiovascular disease [5], and late-life cognitive impairment [6]. At the molecular level, coffee contains bioactive compounds, such as caffeine, polyphenols, pyrroles, and furans [4,7]. Among them, caffeine is a well-known compound that displays pharmacological activity and has been employed in some drugs as a psychoactive or central nervous system (CNS) stimulant [8]. Caffeine has been widely used in pharmacological examination and displays effects on obesity, cardiovascular diseases, and memory consolidation [9,10]. However, the safety pharmacological action of other coffee components has not been investigated.

Recently, chlorogenic acid (CGA), which mainly contains 5-*O*-caffeoylquinic acid (5-CQA) in coffee, has received much attention due to its pharmacological activity (e.g., antioxidant, hepatoprotective, cardioprotective, anti-inflammatory, neuroprotective, anti-obesity, anti-hypertension, and anti-cognitive dysfunction) [7,11,12,13,14]. For instance, Mills et al. (2017) reported that human endothelial function was improved by coffee intake in a 5-CQA dose-dependent manner [15]. Kato et al. (2018) showed that cognitive function in elderly subjects with complaints of subjective memory loss could be improved by a 6-month intake of CGA [16]. CGAs also contribute to anti-inflammatory activity in C57BL/6 mice with dextran sulfate sodium-induced colitis [17]. Furthermore, CGA could contribute to the improvement of metabolic disorders (hepatic steatosis, cardiovascular disease, diabetes, and obesity), and may display hepatoprotective effects, gut-protective effects [13,18,19,20,21], and chemo-sensitizing effect for the suppression of tumor progression [22,23]. Considering these situations, pharmaceutical use of CGAs might accelerate in the near future and therefore, safety pharmacological evaluation of CGA may become increasingly important.

**Figure 1:**
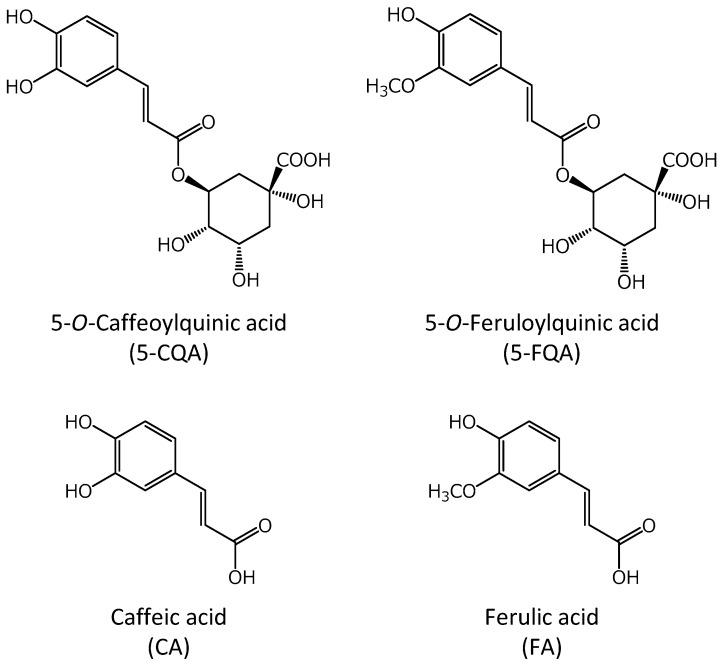
Structures of the chlorogenic acids (CGAs) evaluated in this study.

Therefore, the purpose of this study was to evaluate the safety pharmacological effects of the coffee component, 5-CQA, and its metabolites, 5-*O*-feruloylquinic acid (5-FQA), caffeic acid (CA), and ferulic acid (FA), for the development of pharmaceutical products via high-degree application of coffee components (Figure 1). The guideline on safety pharmacology studies for human pharmaceuticals (ICH S7A) states that compounds should be assessed for core battery functions and functions related to the assumed therapeutic target at concentrations at or above the therapeutic concentration [24]. Hence, corresponding effects of 5-CQA and its metabolites were evaluated using Langendorff perfused heart assay, electrophysiological assay of acute rat hippocampal slices, and in vitro Magnus assay of intestinal tracts. To our knowledge, this is the first study to evaluate the safety pharmacological action of coffee components, except for caffeine. The results obtained will therefore facilitate the design of safety pharmaceutical products with coffee components.

## 2. Results

### 2.1. Langendorff Perfused Heart Assay

**Figure 2:**
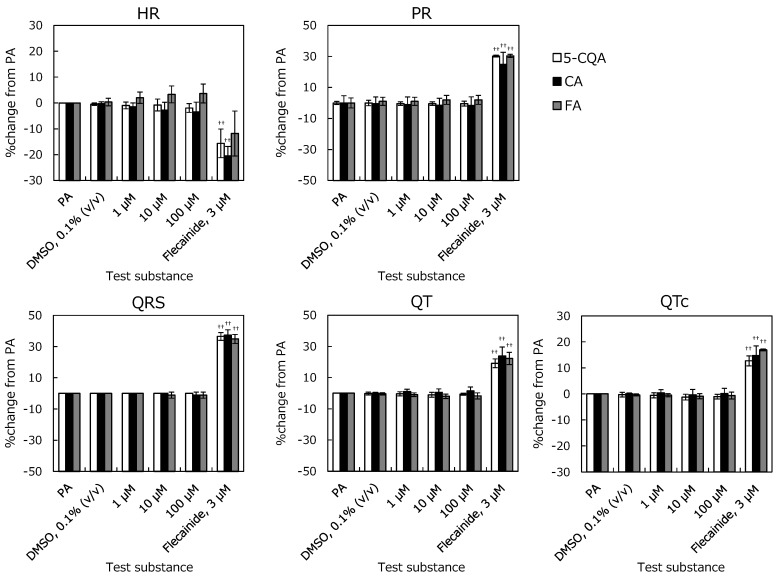
Relationships between electrocardiogram parameters and accumulation of each perfusate. The isolated perfused guinea pig heart was respectively conditioned with 0.1% dimethyl sulfoxide (DMSO), 1–10–100 µM of 5-*O*-caffeoylquinic acid (5-CQA), caffeic acid (CA), and ferulic acid (FA), and 3 µM of flecainide. Heart rate (HR), PR, QRS, QT, and QTc values are represented as % change from Pre-application (PA). Each error bar represents mean ± SD. (n = 3). ^††^: *p* < 0.01; Significantly different from DMSO (Student’s t-test).

**Figure 3:**
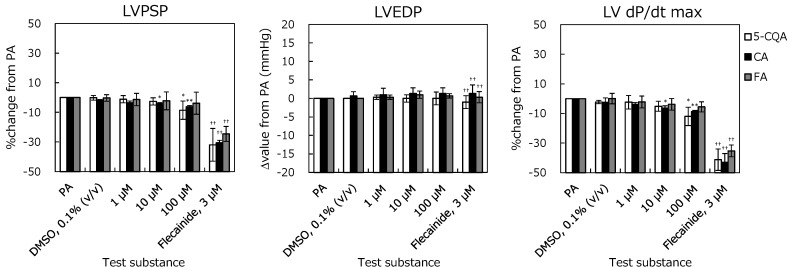
Relationships between left ventricular pressure (LVP) parameters and concentrations of 5-CQA, CA, and FA in the isolated perfused guinea pig heart system. Left-ventricular Peak Systolic Pressure (LVPSP), Left-ventricular End Diastolic Pressure (LVEDP), and left-ventricular-pressure rise (LV dP/dt max) are represented as % change from PA. Each error bar represents mean ± SD (n = 3). *: *p* < 0.025, **: *p* < 0.005; Significantly different from DMSO (Williams test). ^††^: *p* < 0.01; Significantly different from DMSO (Student’s t-test).

The electrocardiogram (ECG) with PR interval (PR), QRS duration (QRS), QT interval (QT), and QTc interval (QTc) and left-ventricular pressure (LVP) with Left-ventricular Peak Systolic Pressure (LVPSP), Left-ventricular End Diastolic Pressure (LVEDP), and left-ventricular-pressure rise (LV dP/dt max) are shown in Figure 2 and Figure 3. The positive-control prolonged PR, QRS, QT, and QTc, and reduced cardiac function. Hence, these changes sufficiently demonstrate the effects of flecainide at 3 μM and validate this study.

For the ECG parameters, no significant change was observed until 100 µM in 5-CQA, CA, and FA compared to solvent control. 5-CQA did not affect the LVP up to 10 μM. At 100 μM, % changes in LVPSP and LV dP/dt max were −8.5 ± 6.2% and −12.0 ± 6.2%. These changes reached statistical significance; however, they were not considered test-substance related because they were in the normal range of the background data. CA did not affect the LVP at concentrations of up to 1 μM. The % changes in LVPSP and LV dP/dt max at 10 μM were −4.3 ± 0.8% and −6.5 ± 1.6%, and those at 100 μM were −6.2 ± 0.9% and −8.8 ± 0.8%, respectively. At these concentrations, statistically significant decrease was noted in LVPSP and LV dP/dt max compared to pre-application. However, these were not considered test-compound related because the % change was very small, and they were in the normal range of the background data. FA did not affect the LVP at the concentration of 100 μM. These results show that 5-CQA, CA, and FA do not affect cardiac function in the Langendorff perfused heart assay.

**Figure 4:**
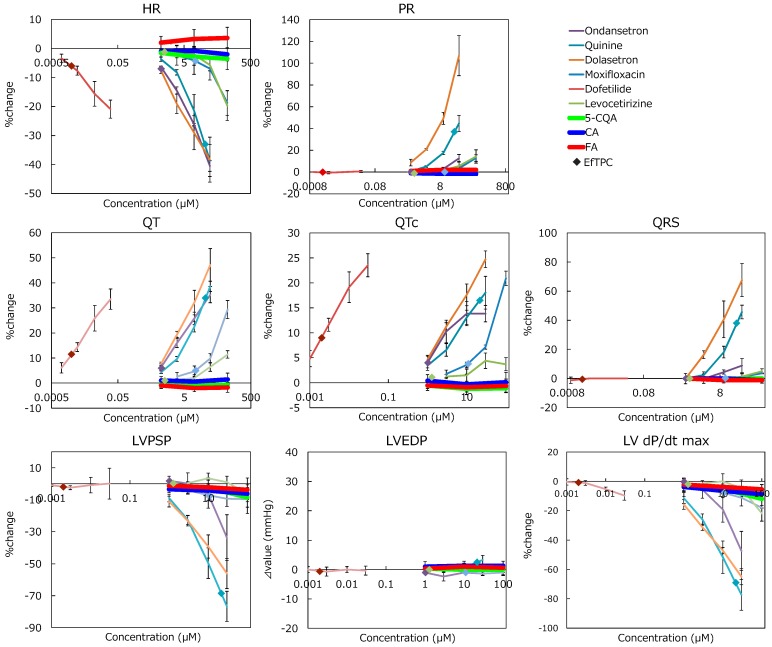
Comparison between drugs with cardiovascular effects and chlorogenic acid (CGA) in the parameters of the Langendorff perfused heart assay. Each error bar represents mean ± SD (n = 3). ◆: depicts effective free therapeutic plasma concentration (color corresponds to each drug). CGA’s effect on each parameter was weaker than that of the drugs.

Alterations by CGAs were compared to drugs. All cardiotoxic drugs affected ECG and LVP parameters near effective free therapeutic plasma concentrations (EfTPC), and Ondansetron, Quinine, and Moxifloxacin altered the parameters below EfTPC (Figure 4 and Table 1). The comparison between drug effects and solvent control effects are provided as supplementary data. Adverse effects were not detected at EfTPC in Dofetilide and Levocetirizine because the applied concentrations were higher than EfTPC. Maximum % change within the existing concentration range of each drug was confirmed as −47.4% in LV dP/dt max (Ondansetron), −77.3% in LV dP/dt max (Quinine), 106.9% in PR interval (Dolasetron), 29.3% in QT interval (Moxifloxacin), 33.5% in QT interval (Dofetilide), and −21.8% in LV dP/dt max (Levocetirizine). Compared to the effects of these cardiotoxic drugs, CGAs showed minimal effects (e.g., at the max, −12% in LV dP/dt max (5-CQA) even at 100 µM) though EfTPC was not determined, implying that these alterations would not be considered to be test-substance related.

### 2.2. Electrophysiological Assays of Rat Hippocampal Slices

#### 2.2.1. Epileptiform Discharges (ED) Rate

**Figure 5:**
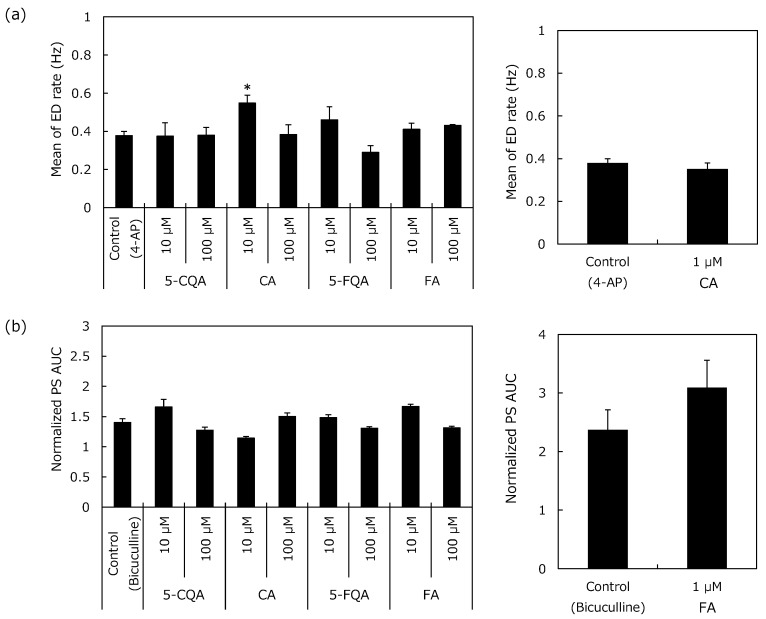
Effects of the chlorogenic acids on the electrophysiological activity of hippocampal slices with convulsive compounds. (**a**) ED rate with 10 µM 4-aminopyridine and (**b**) Population spikes (PS) area under the curve (AUC) with 1 µM bicuculline. (**a**) Amplitudes higher than 15 μV were counted as ED and frequency was averaged by 30-s time slots. The frequency of ED for each condition was plotted (± SEM). (**b**) PS AUCs (between 2 and 100 ms after stimulus) were calculated for each electrode of each slice. Normalized PS AUCs were averaged by slices and PS mean area (± SEM) was plotted. Results represent mean of the last 2 min of application. *p < 0.05 to control by Sidak’s test.

The results after co-exposure to 4-aminopyridine (4-AP) are shown in Figure 5(a). When applied alone at 10 μM, all CGAs did not trigger ED over a 35-min period (0.0 ± 0). Similarly, subsequent co-exposure with 50 μM 4-AP did not alter ED rate from 4-AP control slices, except for CA (5-CQA: 0.38 ± 0.04, FA: 0.41 ± 0.04, and 5-FQA: 0.46 ± 0.05 to control slices: 0.38 ± 0.02 Hz). The ED rate during the 10 µM CA co-exposure with 4-AP was higher than that observed with the 4-AP control slices (0.55 ± 0.07 Hz compared to 0.38 ± 0.02 Hz, *p* = 0.02). A follow-up test was conducted with 1 µM CA but this did not trigger ED over a 30-min period and did not alter ED rate in the 4-AP control slices in the co-exposure period (0.35 ± 0.03 to 0.38 ± 0.04 Hz). 

#### 2.2.2. Population Spikes (PS) Recording

The results after co-exposure with bicuculline are shown in Figure 5b. Bicuculline (1 μM) largely increased PS area under curve (AUC); normalized AUC was 1.40 ± 0.06 after a 40-min application period (mean of the last 2 min of application). When applied alone at 10 μM, all CGAs did not substantially modify PS AUC and spike number over a 30-min period. Normalized AUCs were 1.01 ± 0.04, 1.01 ± 0.02, 1.04 ± 0.04, and 0.99 ± 0.02 for 5-CQA, CA, FA, and 5-FQA, respectively (versus 1.00 ± 0.01 in control periods). Subsequent co-exposure with 1 μM bicuculline increased the PS AUC with lower alterations in CA and higher in FA than those observed in control bicuculline slices (AUCs were 1.15 ± 0.02 in CA and 1.67 ± 0.04 in FA compared to 1.40 ± 0.06 in control slices). The alterations after co-exposure with 1 µM bicuculline were in the same range as the bicuculline control slices in 5-CQA and 5-FQA.

For the additional spike number, a single stimulation triggered multiple spikes in the presence of 1 μM bicuculline (1.32 ± 0.32 additional spike). The additional spike number of 5-CQA, CA, and 5-FQA co-exposure with 1 μM bicuculline was in the same range as the bicuculline control slices (data not shown). FA, however, increased the additional spike number triggered by 1 μM bicuculline via co-application (1.44 ± 0.33 additional spike).

A follow-up test was conducted for 1 µM FA. Normalized AUC was 1.09 ± 0.06 after the single application period (versus 1.00 ± 0.02 in control period) and co-exposure with 1 μM bicuculline increased PS AUC, which was in the same range as that of the observed bicuculline control slices (AUC was 3.09 ± 0.47 compared to 2.37 ± 0.34 in control slices). For the additional spike number, a single stimulation did not trigger multiple spikes for the single application period, but subsequent co-exposure with 1 μM bicuculline triggered 1.63 ± 0.31 additional spikes, which was in the same range as the control bicuculline slices (1.47 ± 0.18 additional spikes).

Although 10 µM CA increased ED rate of 4-AP and 10 µM FA increased PS AUC and the additional spike number of bicuculline, dose dependency was not confirmed, and no significant change was observed at 1 µM. Moreover, all CGAs did not exhibit an adverse effect during the single application period. These results indicate that the risk of CGAs on CNS activity is negligible.

### 2.3. In Vitro Magnus Assay

**Figure 6:**
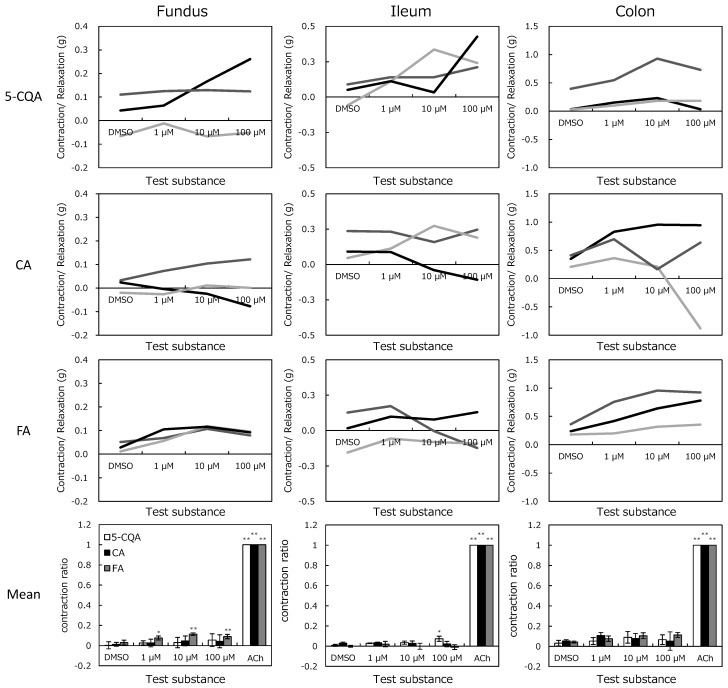
Concentration-response curves of chlorogenic acids from the fundus, ileum, and colon isolated from guinea pig. Test substances were applied to organs after a stabilization period. Values represent the exchange of isolated organ tension compared to before dimethyl sulfoxide (DMSO) application. Mean represents mean ± SEM of the contraction normalized to 20 µM acetylcholine (ACh) in the fundus and 0.2 µM ACh in the ileum and colon. *p < 0.05 and **p < 0.01 to DMSO by Dunnett’s test.

The addition of CGAs to the isolated organs caused various responses at each concentration (Figure 6). Figure 6 shows that 5-CQA increased the ileum tension while FA increased tensions in the fundus. The tensions were increased from 0.027 ± 0.044 g to 0.293 ± 0.068 g in the ileum by 100 µM 5-CQA (*p* < 0.05), and 0.030 ± 0.012 to 0.089 ± 0.005 g in the fundus by 100 µM FA (*p* < 0.01). Although other tissue tensions were also altered, these changes differed with the same test compound and concentrations. Nonetheless, because there were no significances, they were not considered as effects of the test substances. Variability in the alteration was considered to be peristaltic movement, although the organ bath containing Tyrode’s solution was maintained at 32 °C for suppression. 

### 2.4. In Vitro Profiling Assay

The interactive ability of CGAs with the targets is shown in Table 2. Targets with key functions related to safety pharmacology, which have been screened at pharmaceutical companies, have been listed previously [28]. Of these, 38 targets related to cardiovascular system (CVS), CNS, and gastrointestinal (GI) functions were assessed. CGAs interacted with all targets assessed with an inhibition less than 30% at 30 µM. Although the results of the electrophysiological assay of rat hippocampal slices indicated that CA interacted with the potassium channel, the 4-AP target, and FA interacted with γ-aminobutyric acid (GABAA), the bicuculline target, neither interactions were detected. The top three interactions (26% Inhibition of FA with Calcium channel L-Type, Phenylalkylamine related to CVS; 22% Inhibition of 5-FQA with Dopamine, D1 related to CVS; and 21% inhibition of CA with Calcium channel L-Type, Phenylalkylamine related to CVS) were not confirmed in any previous research (IC50 < 30 µM) and no effect has been confirmed by ex vivo assays. Ingenuity^®^ Pathway Analysis (IPA, Ingenuity Systems, Qiagen, USA) was used for target search but there was no direct interaction between the proteins assessed and 5-CQA, CA, and FA (5-FQA was not recorded in IPA). Therefore, all CGAs do not possess a high ability to interact with the targets assessed.

## 3. Discussion

We first revealed that no remarkable adverse pharmacological activities of CGAs were discovered using ex vivo assays and in vitro profiling assays. To our knowledge, this is the first report to present a comprehensive evaluation of the safety pharmacology of the components of coffee, except for caffeine. The pharmacological effects on the CVS and CNS, which are related to core-battery evaluation in pharmaceuticals, and GI functions, one of the assumed therapeutic target, were evaluated. As a result, no adverse effect was observed in the Langendorff perfused heart assay. Nonetheless, CA and FA had some pharmacological effects as revealed in the electrophysiological assay of rat hippocampal slices with co-exposure to the convulsive compound. Dose dependency was however not confirmed, and no significant changes were observed at the lower dose (1 µM). Meanwhile, in the Magnus assay, a slight improvement in the contraction activity was observed with FA in the stomach fundus and 5-CQA in the ileum.

In the Langendorff perfused heart assay, CGAs had no effects on CVS functions until 100 µM, which was 100-fold higher than the human plasma concentration of each CGA following the ingestion of 412–795 µmol of CGAs (119–373 µmol of 5-CQA) [29,30]. On the other hand, cardiotoxic drugs affected CVS functions around EfTPC. The largest margin between the lowest concentration indicated a statistically significant change and at most, EfTPC was less than 50-fold (50 µM (lowest concentration indicating statistically significant change)/1 µM (EfTPC)) of Dofetilide. These results indicated that the risk of CGAs on CVS functions could be considered to be quite low. 

Although a remarkable adverse effect was not identified in the electrophysiological assay, some sporadic effects, such as increasing ED rates of 4-AP and PS AUC of bicuculline in CA and FA, were observed at 10 µM. However, human plasma concentration of each CGA following the ingestion of coffee was less than 1 µM [29,30]. Second, for CGAs to reach the CNS, penetration via the blood brain barrier is required. Although some researchers used an in vitro model to show that polyphenol could penetrate the blood brain barrier [31,32], the permeability rate remains unclear. In general, hydrophilic compounds such as CGAs are difficult to penetrate the blood brain barrier, indicating that the permeability rate of CGAs would be low. Therefore, the concentration of CGAs in the human brain would not reach 10 µM, even if a relatively high amount of CGAs (ca. 10–100 times higher than food experience) is used in pharmaceuticals (the type of drugs and route of administration are not considered).

For the effects on GI functions, the review reports available on the Pharmaceuticals and Medical Devices Agency website (https://www.pmda.go.jp/) show that drugs that adversely affect GI functions reduced peristaltic movement at a lower concentration (for instance, Lamotrigine (an anticonvulsant drug) reduced this movement at 0.3 µM). Compared to acetylcholine, which contracts GI smooth muscle, alterations by CGAs were less than 15% even at 100 µM. Furthermore, a slight contraction activity might be considered as an indicator of an enterokinesis promoting agent. In fact, Badary et al. (2006) reported a finding similar to the present study that FA significantly accelerates GI transit and gastric emptying in rats in a dose-dependent manner [33]. Moreover, they interpreted this result that FA may help to avoid the adverse event of drug-induced delay in gastric emptying. Therefore, the results of the present Magnus assay would indicate enterokinesis promotion rather than adverse effects.

In in vivo studies, according to a review of toxicological literature of 5-CQA and CA published from National Toxicology Program [34], no pharmacological adverse effects have been reported from short, sub-chronic, and chronic toxicity tests; however, these studies did not focus on the safety pharmacological effects. For instance, chronic exposure of mice for 96 weeks and rats for two years to CA revealed no safety pharmacological toxicity [35]. These results support the ex vivo assay results that no clear adverse effect was identified.

## 4. Materials and Methods

### 4.1. Chemical Agents

5-CQA, CA, and FA were purchased from Sigma-Aldrich (Missouri, USA). Caffeine was purchased from Alfa Aeser (Lancashire, UK). 5-FQA was refined from green coffee extract in our laboratory by reported methods with a slight modification [36]. Briefly, ground green-coffee beans were extracted with hot water, and the extract was reduced to a powder with the spray-dry method. The extract dissolved in water was applied to an aromatic-type adsorbent column (Sepabeads SP70; Mitsubishi Chemical aqua solutions, Tokyo, Japan) and CGA mixture was eluted with 0.1% NaOH. Then, the quinic acid derivatives (including 5-CQA and 5-FQA) were isolated using a medium pressure chromatography system (Yamazen, Osaka, Japan) equipped with an Ultra Pack ODS-A-40D column, UV detector PREP-UV-10V, fraction collector FR 50N, gradient mixer GR200, degasifier, and pump PUMP-60A. 5-FQA were further separated using a preparative high-performance liquid chromatography (HPLC) system (Interface PLC 561, Pump PU 715, Column oven CO 705, UV detector UV 702, fraction collector FC 204, Auto sampler MIDAS, degasifier GASTORR722, GL Science, Inc., Tokyo, Japan) equipped with an Inertsustain C18 column (GL Science, Inc., Tokyo, Japan) using trifluoroacetic acid–acetonitrile–water (1:120:880) as the eluent at a flow rate of 9 mL/min. The elution pattern was monitored by measuring the absorbance at 325 nm and the purity of 5-FQA was analyzed (> 98%). All other chemicals used were of reagent grade and were purchased commercially. 

### 4.2. Ex Vivo Assays

All animal assays in this study were approved by the Animal Care Committee of the Kao Corporation and contract research organizations where each assay was conducted (see acknowledgement). 

#### 4.2.1. Langendorff Perfused Heart Assay

Male Hartley guinea pigs (weight, 571 to 674 g; Japan SLC, Inc., Shizuoka, Japan) were anesthetized by inhalation of 5% isoflurane (isoflurane for animal, Mylan Inc., Georgia, GA, USA) and a mixture consisting of oxygen and nitrous oxide gas at a ratio of 3:7. A tracheotomy cannula was inserted under anesthesia with 1% to 2% isoflurane and the gas mixture by an artificial-respiration device (5 mL/body; 50 times/min; SN-480–7; Shinano Inc., Tokyo, Japan). After injection of heparin sodium (100 unit/body, AY Pharmaceuticals Co., Ltd.) into the jugular vein, the animals were exsanguinated. The hearts were excised and immediately placed in ice-cooled Krebs-Henseleit (KH) solution containing NaCl 118 mM, KCl 4.7 mM, CaCl_2_·2H_2_O 2.5 mM, MgSO_4_·7H_2_O 1.2 mM, KH_2_PO_4_ 1.2 mM, NaHCO_3_ 25 mM, and D(+)-glucose 11.1 mM. A stainless-steel cannula was inserted in the aorta, followed by attachment to the Langendorff apparatus (Physio-Tech Co., Ltd., Tokyo, Japan). The attached heart was perfused with KH solution at 37.0 ± 0.2 °C. Before use, the KH solution was aerated by a gas mixture (95%O_2_ + 5%CO_2_) for 30 min or longer. The perfusion pressure was maintained at 70 mmHg.

5-CQA, CA, and FA were dissolved in DMSO. DMSO solutions at 1, 10, and 100 mM were diluted 1000-fold with the KH solution to make test solutions of 1, 10, and 100 μM, respectively. The test solutions were cumulatively applied at a flow rate of 20 mL/min for 10 min with each concentration.

An ECG signal was recorded using electrodes fixed on the ventricular apex (+), and a stainless-steel cannula was inserted in the aorta (−), and LVP was recorded from the balloon catheter placed in the left ventricle. The ECG signals were amplified with an amplifier (DAM50; World Precision Instruments, Inc.) and LVPs were monitored with a Pressure Monitor 4 (Living Systems Instrumentation). Both of the waveforms were recorded with computer software (1000 Hz; iox2 and ecgAUTO3; emka Technologies).

**Figure 7:**
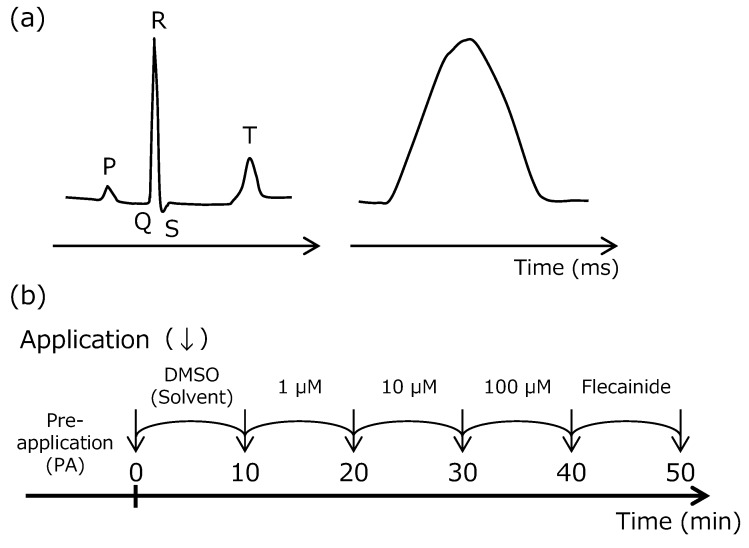
Parameters recorded from the Langendorff perfused heart assay. (**a**) One wave shape of electrocardiogram (ECG) and left ventricular pressure (LVP) recording. The letters represent peak name on the ECG. (**b**) The standard protocol of this assay. 5-*O*-caffeoylquinic acid (5-CQA), caffeic acid (CA), and ferulic acid (FA) were applied at 1, 10, and 100 µM, cumulatively. The recording period of one condition was 10 min at each concentration of the test substance. The parameters for control conditions were recorded before and after test substances (solvent control, 0.1% DMSO; Positive control, 3 µM flecainide).

The flow of the assay is shown in Figure 7. Before measurement, the in-balloon pressure was adjusted so that the diastolic blood pressure would be in the range of 0 to 10 mmHg. The cardiac parameters, such as HR, PR interval, QRS duration, QT interval, QTc interval (Fridericia’s formula: QTc = QT/RR^1/3^), LVPSP, left-ventricular end diastolic pressure (LVEDP), and LV dP/dt max, were measured. 

Data are expressed as mean (± SD). The measured % changes from the pre-application value were used for analyses. Williams test was carried out between the solvent-control and test compound. Student’s t-test was carried out between the solvent-control and positive-control. The laboratory background data of drugs, of which the adverse effects on CVS were reported in SIDER database (http://sideeffects.embl.de/), were compared to the results of CGAs to interpret the significance of the alternations. The data from the solvent control and drug groups were tested by F test for homogeneity of variance. F test was performed at the significance level of 5%. When the variances were homogeneous, Student’s t-test was subsequently performed. When the variances were nonhomogeneous, Welch t-test was selected. The lowest concentrations indicating statistically significant % change to solvent control and EfTPC of each drug are shown in Table 1.

#### 4.2.2. Electrophysiological Assays

##### Preparation of the Hippocampal Slices 

All animals were housed and used in accordance with the French and European legislations for animal care. Rats (Janvier Labs, Le Genest-Saint-Isle, France) were euthanized by fast decapitation, without prior anesthesia. The brain was quickly removed and soaked in ice-cold oxygenated buffer containing KCl 2 mM, NaH_2_PO_4_ 1.2 mM, MgCl_2_ 7 mM, CaCl_2_ 0.5 mM, NaHCO_3_ 26 mM, glucose 11 mM, and sucrose 250 mM. Hippocampal slices (400 μm) were cut with McILWAIN tissue chopper and quickly incubated at room temperature for at least 1 h in artificial cerebrospinal fluid containing NaCl 126 mM, KCl 3.5 mM, NaH_2_PO_4_ 1.2 mM, MgCl_2_ 1.3 mM, CaCl_2_ 2 mM, NaHCO_3_ 25 mM, and glucose 11 mM. During the experiments, slices were perfused with oxygenated artificial cerebrospinal fluid at 37 °C at a rate of 3 mL/min. The tested slice numbers were > 2 for 100 µM, > 4 for 10 µM, and > 6 for 1 µM (follow-up study). All experiments were carried out with 3-dimensional multielectrode array (Qwane Biosciences S.A., Lausanne, Switzerland) and data were recorded with a commercially available multielectrode array set-up from MultiChannel Systems (MCS GmbH, Reutlingen, Germany).

##### ED Recording

A hippocampal slice was placed on the multielectrode array (200 μm distant electrodes) to ensure the multi-electrode grid covered the major surface of the slice. After a 5-min control recording to verify the absence of spontaneous activity, test compound was applied at 10 μM or 100 μM for 30 min (CA was applied at 1 µM for the follow-up study). Then, 10 μM 4-AP, a selective potassium channel blocker, was applied in the continuous presence of the compound for an additional 40-min. Control 4-AP slices were recorded in parallel (following 35 min of control recording, 10 μM 4-AP was applied for a 40-min period) to compare the induction of ED with 4-AP alone and in combination with the compound.

ED frequency (in Hz) was determined as follows: raw data were filtered with a Low Pass filter (Butterworth second order filter) set to 20 Hz. ED number was monitored on-line (epileptiform event amplitudes had to be higher than 15 μV to be counted; a dead time of 200 ms was applied for each epileptiform event detected). ED frequency was averaged using 30-s time intervals. Results are presented as mean frequency of ED for the last 2 min of each condition (± SEM). Statistical significance was measured using two-way ANOVA analysis with Sidak’s multiple comparisons test, except for 100 µM because of its small slice number.

##### PS Recording

A hippocampal slice was placed on the multielectrode array (100 μm distant electrodes). One electrode was chosen to stimulate schaffer collaterals (SC) at the CA1 border. The stimulus in a monopolar biphasic current pulse (−300 μA for 60 μs followed by +300 μA for 60 μs) was applied at 30-s intervals. An Input/Output (I/O) curve was derived before each experiment to determine the stimulation intensity. Basal stimulation intensity was then set to 100 μA higher than the minimal intensity necessary to induce PS in the pyramidal cell layer.

After 20 min of recording in the control condition, test compound was applied alone at 10 μM or 100 μM for 30 min (FA was applied at 1 µM for the follow-up study). Then, 1 μM bicuculline, the antagonist of GABAA, was applied in the continuous presence of the compound for an additional 40-min. Control bicuculline slices were recorded in parallel and after 50 min of control recording, 1 μM bicuculline was applied for a 40-min period.

AUC of the PS (between 2 and 100 ms after stimulus) was calculated for each electrode of each slice using Igor Pro 6.22 software. For normalization, each PS AUC is expressed as a percent of the mean-averaged area recorded over the 20-min control period (before compound application). Normalized PS AUC was averaged by slices for each experiment carried out in the same conditions. PS AUC (± SEM) is expressed as a function of time. The number of PS oscillations induced at the end of the bicuculline-exposure period was determined for each electrode and averaged by slices. The results are presented as mean of the last 2 min of application. Statistical significance was measured using two-way ANOVA analysis with Sidak’s multiple comparisons test, except for the 100 µM concentration because of its small slice number.

### 4.3. In Vitro Magnus Assay

Guinea pigs (Japan SLC, Inc., Shizuoka, Japan) were anesthetized with pentobarbital sodium (30–50 mg/kg, i.p.) and the stomach fundi, ileums, and colons were dissected after being exsanguinated. Tissues were immediately placed in Tyrode’s solution containing NaCl 136.9 mM, KCl 2.7 mM, MgCl_2_.6H_2_O 1.2 mM, CaCl_2_.2H_2_O 2.0 mM, NaHCO_3_ 11.9 mM, and D(+)-glucose 5.6 mM, and flushed for luminal content removal. Segments of approximately 2 cm were cut from each tissue and placed in an organ bath containing Tyrode’s solution aerated with O_2_:CO_2_ (95:5) at 32 °C. The tissue segments were connected to an isometric transducer (load, 1.0 g) to record the contractions (Nihon Kohden Corporation, Tokyo, Japan).

5-CQA, CA, and FA were dissolved in DMSO and added to organ bath cumulatively (final concentrations were 1, 10, and 100 µM). Maximum recording period for one condition was 20 min, and the test solution was exchanged when no effect was detected within 3 min. After the 100 µM period, acetylcholine was applied to confirm all tissues had a similar mechanical activity (stomach fundus, 20 µM; ileum and colon, 0.2 µM). The concentration of acetylcholine was set based on preliminary experiments. Statistical significance was measured using two-way ANOVA analysis with Dunnett’s multiple comparisons test.

### 4.4. In Vitro Profiling Assays

Binding and functional assays of CGAs with the proteins known to be pharmacological targets related to the CVS, CNS, and GI functions were conducted. Briefly, the purified membrane or protein from recombinant or tissue sources expressing the target, and radioisotope-labeled (binding assay) or non-labeled (functional assay) high-affinity ligand were incubated with 30 µM of the test compound. In the binding assay, we measured the radiation activity of the solution containing filtered proteins that bound to the tested compound or high-affinity ligand, while in the functional assays, we measured the reaction product. The ability of the test compound to interact with the tested targets was measured as the inhibition of ligand binding to the target or reaction product production (% inhibition). The tested targets are summarized in Table 2.

## 5. Conclusions

In conclusion, by using ex vivo and in vitro profiling assays in accordance with ICH S7A guideline, safety pharmacological activities of CGAs were evaluated. Based on the results, we demonstrated that CGAs have fundamental properties for use as safe compounds in safety pharmacology. These results support its usage in pharmaceuticals, although a further study might be needed to clarify its absorption, distribution, metabolism, and excretion (ADME) and homeostasis functions. Adverse events in safety pharmacology are life threatening and are not limited to CGAs; therefore, conducting a pharmacological evaluation of other bioactive compounds [37,38,39] is favorable before marketing to avoid the occurrence of adverse events. We believe that the strategy employed in the present study may contribute to the development of safe pharmaceuticals using bioactive food materials.

## Figures and Tables

**Table 1:** Lowest concentration indicating statistically significant % change to solvent control for assessed parameters and effective free therapeutic plasma concentrations (EfTPC) of each drug (µM).

Drug Name	HR	PR	QRS	QT	QTc	LVPSP	LVEDP	LV dP/dt_max_	EfTPC
Ondansetron	1	10	10	1	1	1	1	1	1.023 [25]
Quinine	1	3	3	1	1	1	30	1	21.577 [25]
Dolasetron	1	1	3	1	1	1	-	1	-
Moxifloxacin	30	30	10	3	3	3	-	100	10.960 [26]
Dofetilide	0.001	0.01	-	0.001	0.001	0.03	-	-	0.020 [27]
Levocetirizine	30	10	100	30	3	10	-	100	1.286 [25]

**Table 2:** Summary of the in vitro profiling assays.

Target Name	Assay Type	Species	Main Organ Class or System	% Inhibition			
				CGA	CA	FQA	FA
Adenosine A2A	B	human	CVS, CNS	−7	4	2	−14
Adrenergic α1A	B	rat	CVS, GI, CNS	14	10	10	−2
Adrenergic α2A	B	human	CVS, CNS	5	−4	−3	−2
Adrenergic β1	B	human	CVS, GI	−1	3	−4	−2
Adrenergic β2	B	human	CVS	1	2	6	2
Calcium Channel L-Type, Benzothiazepine	B	rat	CVS	−3	−3	6	6
Calcium Channel L-Type, Dihydropyridine	B	rat	CVS	1	7	6	1
Calcium Channel L-Type, Phenylalkylamine	B	rat	CVS	14	21	19	26
Cannabinoid CB1	B	human	CNS	−6	−9	−6	−15
Cholecystokinin CCK1 (CCKA)	B	human	GI	10	12	7	1
Cyclooxygenase COX-1	F	human	GI	2	6	11	5
Cyclooxygenase COX-2	F	human	CVS	7	9	18	14
Dopamine D1	B	human	CVS, CNS	7	5	22	−1
Dopamine D2L	B	human	CVS, CNS	7	16	−8	9
Endothelin ETA	B	human	CVS	3	4	1	1
GABAA	B	rat	CNS	8	18	15	1
Glutamate, NMDA	B	rat	CNS	9	5	10	16
Histamine H1	B	human	CVS	−12	−11	−22	−9
Histamine H2	B	human	GI, CVS	0	−6	−5	−5
Monoamine Oxidase MAO-A	F	human	CVS, CNS	−5	0	0	5
Muscarinic M1	B	human	CNS, GI, CVS	0	5	−5	0
Muscarinic M2	B	human	CVS	−1	23	7	17
Muscarinic M3	B	human	GI	5	−3	2	−1
Opiate δ1	B	human	CNS, CVS	5	11	2	16
Opiate κ	B	human	GI, CNS, CVS	−3	3	−7	3
Opiate μ	B	human	CNS, GI, CVS	8	−6	5	4
Peptidase, CTSG (Cathepsin G)	F	human	CVS	8	1	2	−2
Phosphodiesterase PDE4	F	human	CNS	−6	4	2	7
Potassium Channel hERG	B	human	CVS	3	−19	6	7
5-HT1A	B	human	CNS	12	9	11	2
5-HT1B	B	human	CVS, CNS	−1	3	−10	4
5-HT2A	B	human	CVS, CNS	3	7	8	8
5-HT2B	B	human	CVS	−16	1	−5	5
5-HT3	B	human	GI	−9	−5	−3	−13
Sodium Channel	B	rat	CVS	19	1	10	5
Transporter, Dopamine (DAT)	B	human	CNS	−10	2	6	0
Transporter, Norepinephrine (NET)	B	human	CNS, CVS	−3	0	−1	2
Vasopressin V1A	B	human	CVS	0	9	16	6

GABAA, γ-aminobutyric acid type A; NMDA, N-methyl-d-aspartase; 5-HT, Serotonin (5-Hydroxytryptamine) receptor; B, Binding assay; F, Functional assay; CVS, cardiovascular system; CNS, central nervous system; GI, gastrointestinal.

## Supplementary Materials

The following are available online at https://www.mdpi.com/1424-8247/12/3/110/s1, Figure S1: Comparison between drug and solvent control effects in the parameters of the Langendorff perfused heart assay.
