# Supplementary material for: Safety Pharmacological Evaluation of the Coffee Component, Caffeoylquinic Acid, and Its Metabolites, Using Ex Vivo and In Vitro Profiling Assays
Source: Pharmaceuticals (Basel). 2019 Jul 17;12(3):110. doi: 10.3390/ph12030110 (PMC6789782; doi:10.3390/ph12030110)
Supplement: Supplementary file 1 [file pharmaceuticals-12-00110-s001.pdf]

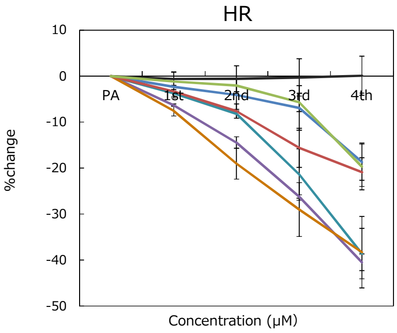

	PA	1st	2nd	3rd	4th
DMSO	0	0.1%	0.1%	0.1%	0.1%
Ondansetron	0	1††	3††	10**	30††
Quinine	0	1††	3††	10††	30††
Dolasetron	0	1††	3††	10††	30††
Moxifloxacin	0	3	10	30††	100††
Dofetilide	0	0.001††	0.003††	0.01††	0.03††
Levocetirizine	0	3	10	30†	100††

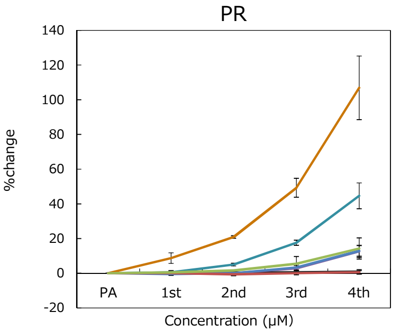

	PA	1st	2nd	3rd	4th
DMSO	0	0.1%	0.1%	0.1%	0.1%
Ondansetron	0	1	3	10†	30*
Quinine	0	1	3††	10††	30**
Dolasetron	0	1*	3†	10**	30**
Moxifloxacin	0	3	10	30††	100*
Dofetilide	0	0.001	0.003	0.01*	0.03
Levocetirizine	0	3	10*	30	100

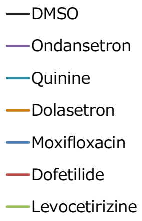

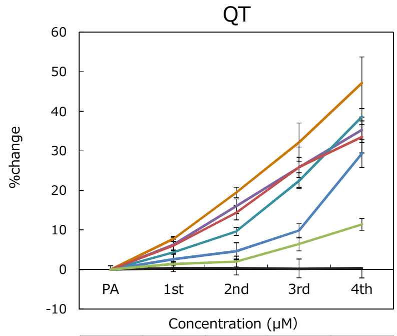

	PA	1st	2nd	3rd	4th
DMSO	0	0.1%	0.1%	0.1%	0.1%
Ondansetron	0	1††	3††	10††	30††
Quinine	0	1††	3††	10††	30††
Dolasetron	0	1††	3††	10**	30**
Moxifloxacin	0	3††	10††	30††	100††
Dofetilide	0	0.001*	0.003††	0.01*	0.03††
Levocetirizine	0	3	10	30††	100††

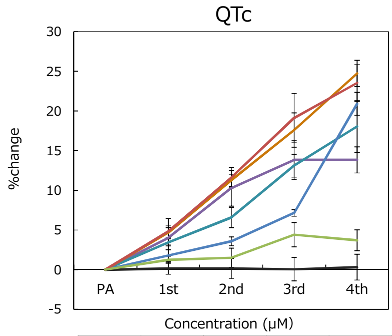

	PA	1st	2nd	3rd	4th
DMSO	0	0.1%	0.1%	0.1%	0.1%
Ondansetron	0	1*	3††	10††	30††
Quinine	0	1††	3††	10††	30*
Dolasetron	0	1††	3††	10††	30††
Moxifloxacin	0	3††	10††	30††	100††
Dofetilide	0	0.001*	0.003††	0.01**	0.03††
Levocetirizine	0	3†	10	30††	100††

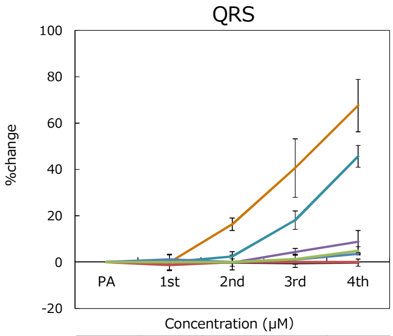

	PA	1st	2nd	3rd	4th
DMSO	0	0.1%	0.1%	0.1%	0.1%
Ondansetron	0	1	3	10††	30
Quinine	0	1	3†	10*	30**
Dolasetron	0	1	3††	10*	30**
Moxifloxacin	0	3	10	30	100**
Dofetilide	0	0.001	0.003	0.01	0.03
Levocetirizine	0	3	10	30	100††

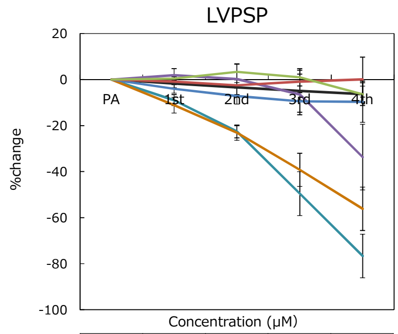

	PA	1st	2nd	3rd	4th
DMSO	0	0.1%	0.1%	0.1%	0.1%
Ondansetron	0	1††	3	10	30**
Quinine	0	1††	3††	10*	30††
Dolasetron	0	1††	3††	10††	30††
Moxifloxacin	0	3†	10	30	100
Dofetilide	0	0.001	0.003	0.01	0.03†
Levocetirizine	0	3	10†	30†	100

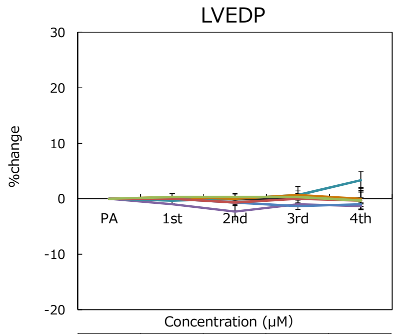

	PA	1st	2nd	3rd	4th
DMSO	0	0.1%	0.1%	0.1%	0.1%
Ondansetron	0	1**	3††	10**	30
Quinine	0	1	3	10	30††
Dolasetron	0	1	3	10	30
Moxifloxacin	0	3	10	30	100
Dofetilide	0	0.001	0.003	0.01	0.03
Levocetirizine	0	3	10	30	100

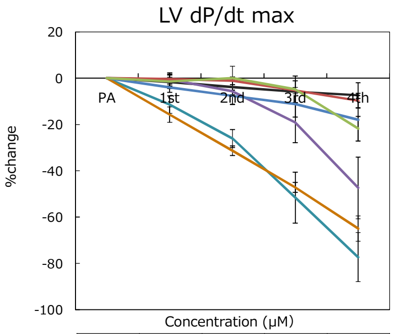

	PA	1st	2nd	3rd	4th
DMSO	0	0.1%	0.1%	0.1%	0.1%
Ondansetron	0	1	3	10††	30*
Quinine	0	1††	3††	10*	30††
Dolasetron	0	1††	3††	10††	30††
Moxifloxacin	0	3	10	30	100††
Dofetilide	0	0.001	0.003	0.01	0.03
Levocetirizine	0	3	10	30	100††

Figure S1. Comparison between drug and solvent control effects in the parameters of the Langendorff perfused heart assay. Each error bar represents mean ± SD (DMSO: n = 39, drugs: n = 3). Drug concentrations were represented in table. *: $p < 0.05$, **: $p < 0.01$; Significantly different from pre-application (PA) (Welch t-test). †: $p < 0.05$, ††: $p < 0.01$; Significantly different from PA (Student's t-test).
